# TGF‐β1 Gene Polymorphisms in Saudi Patients With Type 2 Diabetes With or Without Diabetic Nephropathy

**DOI:** 10.1155/ije/6498033

**Published:** 2026-04-01

**Authors:** Amani Alhozali, Suad Muthaffar, Samar Sultan, Nehad Makki, Nuha Alrayes, Reham Abdulnoor, Ahmed Mirza

**Affiliations:** ^1^ Department of Medicine, Faculty of Medicine, King Abdulaziz University, Jeddah, Saudi Arabia, kau.edu.sa; ^2^ Medical Laboratory Sciences, Faculty of Applied Medical Sciences, King Abdulaziz University, Jeddah, Saudi Arabia, kau.edu.sa; ^3^ Regenerative Medicine Unit, King Fahd Medical Research Center, King Abdulaziz University, Jeddah, Saudi Arabia, kau.edu.sa; ^4^ Princes Al-Jawhara Center of Excellence in Research of Hereditary Disorders, King Abdulaziz University, Jeddah, Saudi Arabia, kau.edu.sa

## Abstract

**Background:**

Diabetic nephropathy (DN) is a major complication of Type 2 diabetes (T2D). Transforming growth factor‐beta 1 (TGF‐β1), a profibrotic cytokine, promotes fibrosis in DN, and its expression is affected by gene polymorphisms. This study aimed to determine the genotypic and allelic frequencies of the *TGF-β1* gene (rs1800469 and rs1800470) as well as to test whether there is an association between *TGF-β1* single‐nucleotide polymorphisms (SNPs) and DN development in Saudi patients with T2D.

**Methods:**

This case–control study involved 204 samples, including 76 controls, 81 patients with T2D without DN, and 47 patients with T2D with DN. Genotyping was performed for two *TGF-β1* SNPs using real‐time PCR and TaqMan. Sanger sequencing was used for validation. Chi‐square testing was conducted to confirm the strength of the association between genotype and allele frequencies and the risk of developing T2D and DN.

**Results:**

The distribution of rs1800469 and rs1800470 polymorphisms was not significantly different between the participants with T2D and control participants. No significant difference was observed in the same SNPs when comparing participants with DN and control participants. The albumin level in patients with DN with the A/A genotype was the highest, whereas it was the lowest in patients with the G/G genotype of *TGF-β1* rs1800470.

**Conclusions:**

*TGF-β1* rs1800470 and albuminuria are significantly associated with DN. *TGF-β1* rs1800469 and rs1800470 were not significantly associated with T2D development and DN progression in Saudi patients with diabetes. Our findings suggest that continued investigation is necessary to understand the underlying mechanisms of *TGF-β1* rs1800470 genotypes in DN pathogenesis.

## 1. Introduction

Type 2 diabetes (T2D) is a serious global public health issue and a prominent cause of healthcare expenditure, mortality, and morbidity [1]. The global prevalence of T2D is growing rapidly in relation to lifestyle changes [2]. T2D is a metabolic disorder characterized by chronic hyperglycemia, caused by decreased insulin secretion or insulin resistance [3]. In Saudi Arabia, which has a population of approximately 34.8 million, 4.27 million individuals have diabetes, of whom 1.86 million are estimated to have undiagnosed diabetes [4]. Diabetic nephropathy (DN) is a common microvascular complication of T2D and develops in approximately 40% of patients with T2D [5]. DN is the worldwide leading cause of chronic kidney disease and end‐stage renal disease (ESRD) [6]. T2D and DN are multifactorial disorders that are caused by genetic variants that interact with environmental factors, such as obesity, smoking, and lifestyle [7, 8].

Transforming growth factor‐β1 (TGF‐β1) cytokine contributes to the development of fibrosis in patients with DN [9]. TGF‐β1 cytokines are the most effective and widespread profibrogenic mediators [10] and promote the synthesis and deposition of numerous extracellular matrix (ECM) proteins, including Collagen I, III, IV, VII, and XVI, in various tissues and cells including the kidney [11]. Furthermore, TGF‐β1 acts as an anti‐inflammatory cytokine that suppresses macrophage activity [12]. Moreover, clinical data show that TGF‐β is instrumental in ESRD via glomerulonephritis [13] and interstitial fibrosis [14]. Hyperglycemia and hyperinsulinemia increase *TGF-β1* expression in mesangial, epithelial, and tubular cells in DN [15]. *TGF-β1* expression and regulation can also be affected by various SNPs, including rs1800469 and rs1800470 [16], which we focus on in this study. The role of these genetic factors in Saudi patients with T2D and with and without DN remains poorly understood.

A Chinese study showed that *TGF-β1* rs1800470 is associated with an increased risk of T2D [17]. Another study indicated an association between *TGF-β1* rs1800470 polymorphisms and T2D with and without DN [18]. The *TGF-β1* rs1800470 polymorphism was also associated with the T2D development and progress of associated DN [16]. Another study on DN by Raina et al. discovered that the CC genotype of *TGF-β1* rs1800470 increased the risk of DN by 3.1–4.5‐fold in Indian populations [19]. Several studies have confirmed that the *TGF-β1* rs1800469 variation is vital for DN [20]. Thus, in this study, we investigated the potential association between the *TGF-β1* rs1800469 and rs1800470 gene polymorphisms in Saudi patients with T2D and with or without DN.

## 2. Materials and Methods

### 2.1. Participants

This is a case–control research study. The study was conducted in the endocrinology clinic and Department of Genetic Medicine, Faculty of Medicine, in association with Princess Al‐Jawhara Centre of Excellence in Research of Hereditary Disorders, King Abdulaziz University, Jeddah, Saudi Arabia, during 2021–2023. Participants (*n* = 204) were selected based on Cochran’s formula, confidence interval (CI) 90%, and a margin of error of 5%, with an estimated proportion of the population with gene polymorphisms of 40%. Individuals were distributed into three different groups: control participants (*n* = 76), patients with T2D without nephropathy (*n* = 81), and patients with T2D and nephropathy (*n* = 47). The study was conducted in accordance with the Declaration of Helsinki (1964) and approved by the Biomedical Ethics and Research Committee of King Abdulaziz University, Jeddah, Saudi Arabia (499‐20).

The inclusion criteria were as follows: diagnosed T2D as defined by the American Diabetes Association (fasting plasma glucose ≥ 126 mg/dL/7 mmol/L), 2‐h postprandial plasma glucose ≥ 200 mg/dL (11.1 mmol/L) during an oral glucose tolerance test, or hemoglobin A1c (HbA1c) ≥ 6.5% (48 mmol/mol). The patient should have classic symptoms of hyperglycemia or hyperglycemic crisis, a random plasma glucose ≥ 200 mg/dL (11.1 mmol/L), and diabetic kidney disease. Inclusion criteria, as defined by the American Diabetes Association, were a clinical diagnosis made based on the presence of albuminuria (urine albumin–creatinine ratios [UACR] > 30 mg/g Cr) and/or reduced estimated glomerular filtration rate (eGFR) < 60 mL/min/1.73 m^2^ in the absence of signs or symptoms of other primary causes of kidney damage, age 30–75, and adequate hepatic, pulmonary, and cardiac function EF > 30%.

The exclusion criteria were as follows: individuals with liver cirrhosis or hepatic failure, impaired cardiac function and ejection fraction < 30%, inflammatory disorders such as systemic lupus erythematosus, use of anti‐inflammatory medications such as nonsteroidal anti‐inflammatory drugs or steroids, a history of malignant tumors, and T1DM. Biochemical parameters were obtained from the medical records of the participants.

### 2.2. Genotype Analysis

Whole blood samples (3 mL) were collected into the ethylenediaminetetraacetic acid–containing tubes and stored at −18°C until DNA extraction. The isolation of genomic DNA from each study participant was done using a commercial DNA extraction kit (Magnesia 16 Automated Nucleic Acid Extraction Instrument, London, UK) as per the manufacturer’s instructions.

Genotyping of *TGF-β1* rs1800469 and rs1800470 single‐nucleotide polymorphisms (SNPs) was performed using real‐time PCR (RT‐PCR) with TaqMan allelic discrimination assays (Applied Biosystems, Thermo Fisher Scientific, USA) on the DNA specimens of patients and controls. Each 10 μL reaction mixture, prepared in 96‐well microtiter plates, contained 1 μL genomic DNA, 5 μL TaqMan Genotyping Master Mix (2×), 3.75 μL distilled water, and 0.25 μL TaqMan SNP genotype assay. Thermal cycling was conducted according to the manufacturer’s recommendations, commencing with an initial denaturation/enzyme activation step at 95°C for 10 min. This was followed by 40 cycles, each consisting of denaturation at 95°C for 15 s, and a combined annealing and extension at 60°C for 1 min. To ensure reliability and accuracy of the allele calls, nontemplate controls were included to monitor for contamination, and established positive controls with known genotypes were used to validate assay performance.

Sanger sequencing was used for RT‐PCR validation. Primers for PCR amplification were designed using Primer3Plus software Version 3.3.0. The forward and reverse primers used for TGFB1 rs1800469 were 3′‐ACC​CAG​AAC​GGA​AGG​AGA​GT‐5′ and 3′‐CAG​GGT​GTT​GAG​TGA​CAG​GA‐5′, respectively. For TGFB1 rs1800470, the forward and reverse primers were 3′‐TCT​GCT​GCC​TCA​GCT​ACT​CA‐5′ and 3′‐AAA​GCA​CCC​AAA​TGG​AGA​AA‐5′, respectively. QIAquick PCR Purification Kits were used to purify PCR products. First, a mixture of binding buffer (100 μL) and 20 μL PCR product was prepared and vortexed. This mixture was then placed onto a QIAquick spin column and centrifuged. After replacing the collection tubes, the columns were washed with 750 μL PE buffer and centrifuged again to remove any remaining washing buffer. The pure DNA was eluted with 30 μL elution buffer, incubated, and centrifuged before being labeled and saved for future use. The columns were discarded.

In the second step of Sanger sequencing, a single primer was used in each reaction to produce single‐stranded DNA molecules using a chain termination reaction performed using the BigDye Terminator v3.1 cycle sequencing kit (Thermo Scientific). The reaction included purified PCR products as template DNA (1 μL for every 10 μL final reaction volume), forward or reverse primers, 2 μL of BigDye Terminator kit reaction mixture, and 6 μL of deionized water. The thermocycler settings were as follows: initial denaturation at 96°C for 3 min, followed by 30 cycles of denaturation at 96°C for 30 s, annealing at 57°C–60°C for 30 s, and extension at 72°C for 2 min, ending with a final extension at 72°C for 5 min. The PCR products from this stage were then purified again and analyzed using the ABI 3500 DNA sequence analyzer (Life Technologies, USA).

The cycle sequencing PCR products were purified by adding 10 μL of BigDye XTerminator beads and 45 μL of SAM solution to each sample. The samples were vortexed for 60 min and centrifuged at 14,000 rpm for 2 min. After centrifugation, 10–20 μL of supernatant was transferred to a 96‐well plate, which was sealed and briefly spun down. The plate was then assembled with a plate base and retainer cover. The sequencing readings were obtained using an ABI 3500 Genetic Analyzer, and the raw data files generated were analyzed using BioEdit sequence alignment software (https://www.mbio.ncsu.edu/BioEdit/bioedit.html).

### 2.3. Statistical Analysis

Data entry and analysis were done using the Statistical Package for the Social Sciences software, Version 28. Description of the data was done using frequency and percentage for qualitative variables and the arithmetic mean and standard deviation for numerical continuous variables. Hardy–Weinberg equilibrium (HWE) analysis was performed to determine whether the alleles and genotypes remain constant from generation to generation for comparing real populations and excluding the possibility of natural selection, genetic drift, migration, or nonrandom mating. Analytic statistics were performed using the chi‐square test to compare qualitative variables and Student′s *t*‐test and one‐way analysis of variance to compare the arithmetic means of a continuous variable between two or more different groups, respectively. Multivariate logistic regression analysis was performed to control for confounding effects, and their results were expressed as adjusted odds ratio (OR) and their 95% CIs. Statistical significance was set as *p* values < 0.05.

Estimated statistical power of the study’s samples (*n* = 204) was computed using G∗Power, and results were as follows:

(1) small  f=0.10 0.5050%,medium  f=0.25 >0.9595%large  f=0.40 1.00100%.,

Thus, even if we assume the large effect, the power of the sample to reject a false null hypothesis (Type II error) will be 100% (above the required threshold of 80%) using Cohen’s *f* = 0.17–0.18. Thus, a sample size of 204 provides > 80% power to detect a small‐to‐medium effect (Cohen’s *f* = 0.18) at *α* = 0.05.

## 3. Results

### 3.1. Clinical Characteristics of the Participants

A total of 204 participants were included in the study and distributed into three groups: control participants (*n* = 76), patients with T2D and without nephropathy (*n* = 81), and patients with T2D and with nephropathy (*n* = 47). For the genotype rs1800469, chi‐square value (*χ* ^2^) was 0.022, whereas for the genotype rs1800470, it was 0.254. Thus, the genotype counts are consistent with HWE. According to Table 1, the demographic and clinical characteristics differed significantly between the compared groups regarding their age (*p* < 0.001), dyslipidemia (*p* < 0.001), body mass index (BMI) (*p* = 0.035), systolic blood pressure (*p* < 0.001), glycated hemoglobin percentage (HbA1c; *p* < 0.001), fasting blood glucose (*p* < 0.001), low density lipoprotein (*p* = 0.006), cholesterol (*p* = 0.008), triglycerides (*p* = 0.023), eGFR (*p* < 0.001), creatinine (*p* = 0.002), and UACRs (*p* = 0.001).

**TABLE 1:** Demographic, clinical characteristics, and laboratory findings of the participants (*n* = 204).

	Controls *N* = 76	Type 2 diabetes without nephropathy *N* = 81	Type 2 diabetes with nephropathy *N* = 47	*p* value

Gender “*N* (%)”				
Male (*n* = 77)	30 (39.5)	29 (35.8)	18 (38.3)	
Female (*n* = 127)	46 (60.5)	52 (64.2)	29 (61.7)	0.890^∗^

Nationality “*N* (%)”				
Saudi (*n* = 167)	63 (82.9)	67 (82.7)	37 (78.7)	
Non‐Saudi (*n* = 37)	13 (17.1)	14 (17.3)	10 (21.3)	0.816^∗^

Age (years)	49.3 ± 9.5	57.3 ± 9.7	58.2 ± 7.7	< 0.001^∗∗^

Body mass index (Kg/m^2^) “*n* = 202”	*N* = 74	*N* = 81	*N* = 49	
	30.4 ± 6.6	33.2 ± 7.4	33.2 ± 8.2	0.035^∗∗^

Systolic blood pressure (Mm Hg) “*n* = 202”	*N* = 74	*N* = 81	*N* = 47	
	129.1 ± 16.9	139.9 ± 20.3	144.9 ± 18.6	< 0.001^∗∗^

Diastolic blood pressure (Mm Hg) “*n* = 202”	*N* = 74	*N* = 81	*N* = 47	
	75.8 ± 10.4	74.4 ± 13.9	74.4 ± 11.5	0.734^∗∗^

HbA1c% (*n* = 202)	*N* = 76	*N* = 79	*N* = 47	
	5.2 ± 0.3	8.2 ± 1.7	9.3 ± 2.4	< 0.001^∗∗^

Fasting blood glucose (mmol/l) “*n* = 195”	*N* = 71	*N* = 78	*N* = 45	
	5.0 ± 0.5	8.4 ± 2.6	9.9 ± 3.8	< 0.001^∗∗^

Dyslipidemia (*n* = 202)	*N* = 75	*N* = 80	*N* = 47	
No (*n* = 54)	42 (56.0)	9 (11.3)	3 (6.4)	
Yes (*n* = 148)	33 (44.0)	71 (88.7)	44 (93.6)	< 0.001^∗^

LDL (mmol/l) “*n* = 187”	*N* = 64	*N* = 78	*N* = 45	
	3.4 ± 1.0	2.8 ± 1.2	3.1 ± 1.2	0.006^∗∗^

HDL (mmol/l) “*n* = 171”	*N* = 59	*N* = 69	*N* = 43	0.305^∗∗^
	1.2 ± 0.4	1.1 ± 0.4	1.1 ± 0.3	

Cholesterol “*n* = 196” (mmol/l)	*N* = 71	*N* = 78	*N* = 47	
	4.8 ± 1.0	4.2 ± 1.2	4.4 ± 1.2	0.008^∗∗^

Triglycerides “*n* = 195” (mmol/l)	*N* = 71	*N* = 77	*N* = 47	
	1.3 ± 0.7	1.5 ± 0.6	1.6 ± 0.8	0.023^∗∗^

eGFR (*n* = 197)	*N* = 73	*N* = 79	*N* = 45	
	98.4 ± 19.9	92.2 ± 17.7	79.6 ± 22.1	< 0.001

Creatinine (mg/dL) “*n* = 203”	*N* = 76	*N* = 80	*N* = 47	
	70.6 ± 26.5	68.4 ± 18.5	89.9 ± 58.7	0.002^∗∗^

UACR (mg/g) “*n* = 114”		*N* = 68	*N* = 46	
		12.8 ± 7.7	450.6 ± 1059.3	0.001˚

ALT (units/L) “*n* = 198”	*N* = 71	*N* = 80	*N* = 47	
	23.7 ± 14.8	27.4 ± 14.5	28.9 ± 18.2	0.161^∗∗^

AST (units/L) “*n* = 198”	*N* = 71	*N* = 80	*N* = 47	
	21.1 ± 7.7	22.1 ± 8.6	21.3 ± 8.5	0.743^∗∗^

Albumin (g/dL) “*n* = 199”	*N* = 71	*N* = 81	*N* = 47	
	41.6 ± 4.7	42.4 ± 10.4	41.6 ± 5.5	0.761^∗∗^

*Note:* AST, aspartate aminotransferase; ALT, alanine aminotransferase; HbA1c, hemoglobin A1c.

Abbreviations: EGFR, estimated glomerular filtration rate; HDL, high‐density lipoprotein; LDL, low‐density lipoprotein; UACR, urine albumin creatinine ratio; SD: standard deviation.

^∗^Chi‐square test: ^∗^statistically significant.

^∗∗^One‐way analysis of variance. Results are expressed as mean ± SD.

### 3.2. Differences Between the Levels of *TGF-β1* rs1800469 and rs1800470 Expressions Between the Control Participants and Patients With Diabetes and Without Nephropathy

**FIGURE 1:**
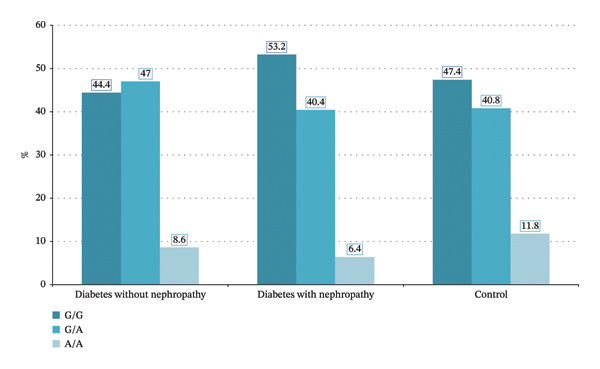
Distribution of TGF‐B1 (rs1800469) in control individuals and patients with diabetes and with and without nephropathy.

**FIGURE 2:**
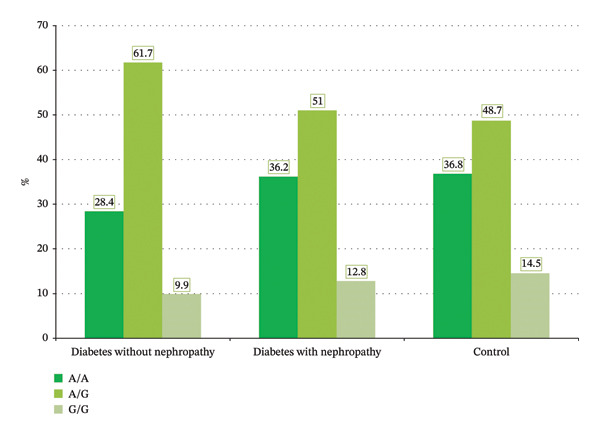
Distribution of TGF‐B1 (rs1800470) in control individuals and patients with diabetes and with and without nephropathy.

Table 2 and Figures 1 and 2 exhibit the insignificant differences between a group of control participants and patients with diabetes and without nephropathy regarding *TGF-β1* rs1800469 and rs1800470, with variable values of OR. Multivariate logistic regression analysis showed that, after controlling for confounders (age, BMI, dyslipidemia, and systolic blood pressure), the differences between control participants and patients with diabetes and without nephropathy were still insignificant (Table 3).

**TABLE 2:** Comparing the *TGF-B1* polymorphisms between the control and patients with diabetic without nephropathy groups.

TGF‐B1	Allele frequency	Genotype frequency	Control subjects	Subjects with Type 2 diabetes without nephropathy	OR (95% CI)	*χ*2	*p* value^∗^
			*N* (%)	*N* (%)			
rs1800469	G (213)		*N* = 76	*N* = 81		0.801	0.670
		G/G (*n* = 72)	36 (47.4)	36 (44.4)	1.0		
	A (101)	G/A (*n* = 69)	31 (40.8)	38 (47.0)	1.23 (0.63–2.38)		
		A/A (*n* = 16)	9 (11.8)	7 (8.6)	0.78 (0.26–2.31)		

rs1800470	A (189)		*N* = 76	*N* = 81		2.75	0.253
		A/A (*n* = 51)	28 (36.8)	23 (28.4)	1.0		
	G (125)	A/G (*n* = 87)	37 (48.7)	50 (61.7)	1.65 (0.82–3.30)		
		G/G (*n* = 19)	11 (14.5)	8 (9.9)	0.89 (0.31–2.57)		

Abbreviations: CI, confidence interval; OR, odds ratio.

^∗^Chi‐square test.

**TABLE 3:** Multivariate analysis to control for confounders between control individuals and patients without diabetic nephropathy.

	**B**	**SE**	**aOR**	**95% CI**	**p** **value**

rs1800469					
G/G^a^			1.0		
G/A	−0.188	0.427	0.83	0.36–1.91	0.660
A/A	−1.088	0.758	0.34	0.08–1.49	0.152

rs1800470					
A/Aa			1.0		
A/G	−0.063	0.458	0.94	0.38–2.30	0.890
G/G	−0.227	0.670	0.80	0.21–2.96	0.735

Age (years)	0.075	0.023	1.08	1.03–1.13	0.001

Dyslipidemia					
No^a^			1.0		
Yes	2.002	0.473	7.40	2.93–18.71	< 0.001

BMI (Kg/m^2^)	0.051	0.031	1.05	0.99–1.12	0.102

Systolic blood pressure (Mm Hg)	0.012	0.012	1.01	0.99–1.04	0.308

*Note:* B: slop, aOR: adjusted odds ratio.

Abbreviation: BMI, body mass index; CI, confidence interval; SE, standard error.

^a^Reference category.

### 3.3. Comparison of the Expression of *TGF-β1* rs1800469 and *rs*1800470 Between the Control Participants and Patients With Diabetes and Nephropathy

Table 4 and Figures 1 and 2 show the insignificant difference in *TGF* *-* *β1* rs1800469 and rs1800470 expression levels between the control participants and patients with diabetes and nephropathy, with an OR approximating to 1, which indicates no effect. Similarly, multivariate logistic regression analysis showed that, after controlling for the confounders (age, BMI, dyslipidemia, and systolic blood pressure), the differences between control individuals and patients with diabetes and nephropathy were still insignificant (Table 5).

**TABLE 4:** Comparison of the *TGF-B1* polymorphisms between the control group and patients with diabetes nephropathy group.

TGF‐B1	Allele frequency	Genotype frequency	Control subjects	Subjects with Type 2 diabetes with nephropathy	OR (95% CI)	*χ*2	*p* value^∗^
			*N* (%)	*N* (%)			
rs1800469	G (172)		*N* = 76	*N* = 47		1.086	0.581
		G/G (*n* = 61)	36 (47.4)	25 (53.2)	1.0		
	A (74)	G/A (*n* = 50)	31 (40.8)	19 (40.4)	0.88 (0.41–1.90)		
		A/A (*n* = 12)	9 (11.8)	3 (6.4)	0.48 (0.12–1.95)		

rs1800470	A (151)		*N* = 76	*N* = 47		0.098	0.952
		A/A (*n* = 51)	28 (36.8)	17 (36.2)	1.0		
	G (95)	A/G (*n* = 87)	37 (48.7)	24 (51.0)	1.07 (0.48–2.36)		
		G/G (*n* = 19)	11 (14.5)	6 (12.8)	0.90 (0.28–2.87)		

Abbreviations: CI, confidence interval; OR, odds ratio.

^∗^Chi‐square test.

**TABLE 5:** Multivariate analysis to control for confounders between control individuals and patients with diabetic nephropathy.

	**B**	**SE**	**aOR**	**95% CI**	***p*** v**alue**

rs1800469					
G/G^a^					
G/A	−0.633	0.561	0.53	0.18–1.59	0.259
A/A	−2.026	1.080	0.13	0.02–1.10	0.061

rs1800470					
A/Aa					
A/G	−0.672	0.601	0.51	0.16–1.66	0.264
G/G	−0.277	0.820	0.76	0.15–3.78	0.736

Age (years)	0.106	0.034	1.11	1.04–1.19	0.002

Dyslipidemia					
No^a^					
Yes	2.782	0.728	16.15	3.88–67.28	< 0.001

BMI (Kg/m^2^)	0.082	0.039	1.09	1.01–1.17	0.033

Systolic blood pressure (Mm Hg)	0.027	0.016	1.03	1.00–1.06	0.079

*Note:* B: slop, aOR: adjusted odds ratio.

Abbreviations: CI, confidence interval; SE, standard error.

^a^Reference category BMI: body mass index.

### 3.4. Comparison of the Expression of *TGF-β1* rs1800469 and rs1800470 Between Patients With Diabetes and With and Without Nephropathy

Table 6 and Figures 1 and 2 show no significant differences between the expression level of *TGF-β1*rs1800469 and rs1800470 in patients with diabetes and nephropathy and those without nephropathy. After controlling for the confounders (age, BMI, dyslipidemia, and systolic blood pressure) in multivariate logistic regression analysis, the differences in *TGF-β1* rs1800469 and rs1800470 expression levels between patients with diabetes and with and without nephropathy remained insignificant (Table 7).

**TABLE 6:** Comparison of the *TGF-B1* polymorphisms between patients with diabetes and with and without nephropathy.

TGF‐B1	Allele frequency	Genotype frequency	Patients with Type 2 diabetes without nephropathy	Patients with Type 2 diabetes with nephropathy	OR (95% CI)	*χ*2	*p* value^∗^
			*N* (%)	*N* (%)			
rs1800469	G (179)		*N* = 81	*N* = 47		0.952	0.621
		G/G (*n* = 61)	36 (44.4)	25 (53.2)	1.0		
	A (77)	G/A (*n* = 57)	38 (47.0)	19 (40.4)	0.72 (0.34–1.53)		
		A/A (*n* = 10)	7 (8.6)	3 (6.4)	0.62 (0.15–2.62)		

rs1800470	A (154)		*N* = 81	*N* = 47		1.387	0.500
		A/A (*n* = 40)	23 (28.4)	17 (36.2)	1.0		
	G (102)	A/G (*n* = 74)	50 (61.7)	24 (51.0)	0.65 (0.29–1.44)		
		G/G (*n* = 14)	8 (9.9)	6 (12.8)	1.01 (0.30–3.47)		

Abbreviations: CI, confidence interval; OR, odds ratio.

^∗^Chi‐square test.

**TABLE 7:** Multivariate analysis to control for confounders between patients with nephropathy and those without it.

	**B**	**SE**	**aOR**	**95% CI**	**p** **value**

rs1800469					
G/G^a^			1.0		
G/A	−0.535	0.416	0.59	0.26–1.32	0.196
A/A	−0.914	0.817	0.40	0.08–1.99	0.263
rs1800470					
A/Aa			1.0		
A/G	−0.619	0.434	0.54	0.23–1.26	0.153
G/G	0.002	0.648	1.002	0.28–3.57	0.998
Age (years)	0.017	0.022	1.02	0.97–1.06	0.458
Dyslipidemia					
No^a^			1.0		
Yes	0.448	0.740	1.57	0.37–6.68	0.454
BMI (Kg/m^2^)	−0.002	0.027	0.998	0.95–1.05	0.934
Systolic blood pressure (Mm Hg)	0.013	0.011	1.01	0.99–1.04	0.256

*Note:* B: slop, aOR: adjusted odds ratio.

Abbreviations: BMI, body mass index; CI, confidence interval; SE, standard error.

^a^Reference category.

### 3.5. Comparison of the Expression of *TGF-β1 rs1800469* and *rs1800470* With Fasting Blood Glucose, Albumin, and HbA1c

Table 8 shows no significant association between levels of albumin, fasting blood glucose, and glycated hemoglobin HbA1c and *TGF-β1*rs1800469 in patients with diabetes and nephropathy. Table 9 shows significant association between *TGF-β1*rs1800470 and albumin levels in patients with diabetes and nephropathy (*p* < 0.05).

**TABLE 8:** Association between *TGF-B1* rs1800469 and levels of albumin, fasting blood glucose, and glycated hemoglobin in patients with diabetes and nephropathy.

	**G/G**	**G/A**	**A/A**	**p**value**^∗^**
	**Mean ± SD**	**Mean ± SD**	**Mean ± SD**	

Albumin (g/dL) (*n* = 47)	*N* = 25	*N* = 19	*N* = 3	0.903
	41.3 ± 5.9	42.1 ± 4.7	41.9 ± 8.0	

Fasting blood glucose (mmol/L) (*n* = 46)	*N* = 24	*N* = 19	*N* = 3	0.946
	9.7 ± 3.8	10.1 ± 3.9	10.0 ± 2.6	

HbA1c% (*n* = 47)	*N* = 25	*N* = 19	*N* = 3	0.759
	9.2 ± 2.1	9.6 ± 2.9	8.7 ± 0.6	

Abbreviation: SD: standard deviation.

^∗^One‐way analysis of variance (ANOVA).

**TABLE 9:** Association between *TGF-B1* rs1800470 and levels of albumin, fasting blood glucose, and glycated hemoglobin in patients with diabetes and nephropathy.

	**A/A *N* = 37 Mean ± SD**	**A/G *N* = 5 Mean ± SD**	**G/G *N* = 1 Mean ± SD**	**p**value**^∗^**

Albumin (g/dL) (*n* = 47)	*N* = 17	*N* = 24	*N* = 6	0.002
	44.3 ± 4.7	41.2 ± 5.0	35.9 ± 4.6	

Fasting blood glucose (mmol/L) (*n* = 46)	*N* = 17	*N* = 23	*N* = 6	0.694
	9.8 ± 4.0	10.2 ± 4.0	8.7 ± 1.7	

HbA1c% (*n* = 47)	*N* = 17	*N* = 24	*N* = 6	0.944
	9.3 ± 2.8	9.5 ± 2.4	9.1 ± 0.8	

Abbreviation: SD, standard deviation.

^∗^One‐way analysis of variance (ANOVA).

The albumin levels of patients with the A/A genotype were the highest, whereas they were the lowest in patients with the G/G genotype of *TGF-β1* rs1800470 (*p* = 0.002). No significant association was detected between the levels of fasting blood glucose and glycated hemoglobin as well as *TGF-β1* rs1800470 in patients with diabetes and nephropathy.

### 3.6. Sanger Sequencing Validation

**FIGURE 3:**
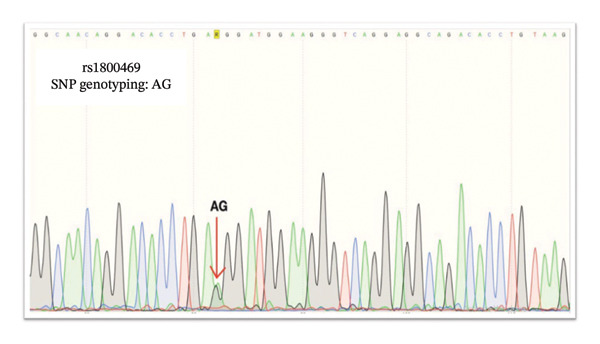
Sanger sequencing result of patients with Type 2 diabetes mellitus (T2D) and rs1800469. AG: heterozygous single‐nucleotide polymorphism as indicated by the arrow.

**FIGURE 4:**
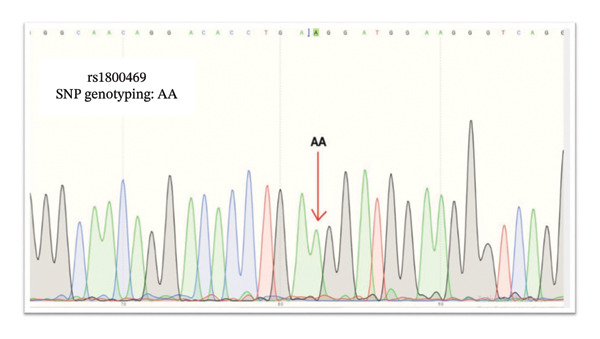
Sanger sequencing result of control individuals with rs1800469. AA: homozygous single‐nucleotide polymorphism as indicated by the arrow.

We used Sanger sequencing to validate our PCR results, indicating that the T2D samples were *TGF-β1* rs1800469 G/A heterozygous, whereas the controls were *TGF-β1* rs1800469 A/A homozygous. Nevertheless, both *TGF-β1* rs1800469 genotypes G/A heterozygous and A/A homozygous were not associated with T2D and DN development in Saudi patients (Figures 3 and 4).

**FIGURE 5:**
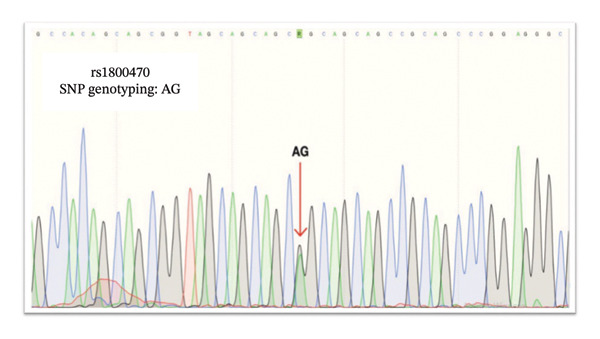
Sanger sequencing result of patients with Type 2 diabetes mellitus (T2D) and rs1800470. AG: heterozygous single‐nucleotide polymorphism as indicated by the arrow.

**FIGURE 6:**
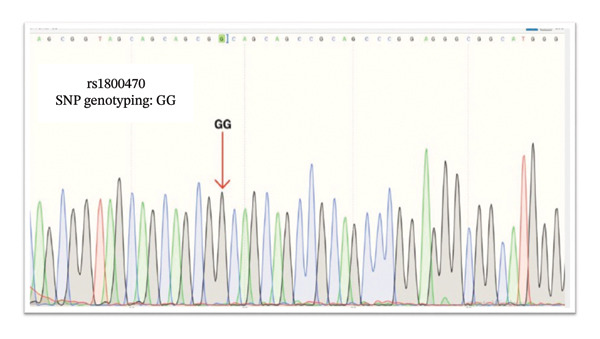
Sanger sequencing result of patients with Type 2 diabetes mellitus (T2D) and rs1800470. GG: homozygous single‐nucleotide polymorphism as indicated by the arrow.

*TGF-β1* gene sequencing indicated that the T2D samples were *TGF-β1* rs1800469 A/G heterozygous (Figure 3); the control samples were *TGF-β1* rs1800469 G/G homozygous (Figure 4). Sanger sequencing validated our results and indicated that T2D samples are *TGF-β1* rs1800470 G/A heterozygous, and the DN samples were *TGF-β1* rs1800470 G/G homozygous. Nevertheless, both *TGF-β1* rs1800470 genotypes G/A heterozygous and G/G homozygous are not associated with T2D and DN development in Saudi patients (Figures 5 and 6).

### 3.7. Comparison of *TGF-β1* rs1800469 and rs1800470 Genotypes Across the Genetic Models

To further investigate potential genetic associations, single‐locus analyses were performed for *TGF-β1* rs1800469 and rs1800470 using codominant, dominant, recessive, overdominant, and log‐additive models. These analyses compared genotype distributions and calculated ORs and 95% CIs in various group comparisons under crude (unadjusted) conditions.

#### 3.7.1. *TGF-β1* rs1800469 Analysis

Control participants vs. T2D (Table 10): No significant associations were found for the *TGF-β1* rs1800469 polymorphism when comparing healthy controls (*n* = 76) against patients with T2D and without nephropathy (*n* = 81) across any of the tested genetic models. For instance, in the codominant model, the G/A genotype showed an OR of 1.23 (95% CI: 0.63–2.38, *p* = 0.670), and the A/A genotype showed an OR of 0.78 (95% CI: 0.26–2.31, *p* = 0.670), both nonsignificant. Similarly, the dominant (OR = 1.13, *p* = 0.713), recessive (OR = 0.70, *p* = 0.508), overdominant (OR = 1.28, *p* = 0.440), and log‐additive (OR = 1.19, *p* = 0.602) models also showed no significant differences.

**TABLE 10:** Single‐locus analysis to detect association between *TGF-B1* rs1800469 and Type 2 diabetes without nephropathy in codominant, dominant, recessive, overdominant, and log‐additive modes: crude analysis.

Model	Genotype	Control individuals *N* = 76 *N* (%)	Patients with Type 2 diabetes without nephropathy *N* = 81 *N* (%)	OR (95% CI)	*p* value
Codominant	G/G	36 (47.4)	36 (44.4)	1.0	0.670
	G/A	31 (40.8)	38 (47.0)	1.23 (0.63–2.38)	
	A/A	9 (11.8)	7 (8.6)	0.78 (0.26–2.31)	

Dominant	G/G	36 (47.4)	36 (44.4)	1.0	0.713
	G/A‐A/A	40 (52.6)	45 (55.6)	1.13 (0.60–2.11)	
Recessive	G/G‐G/A	67 (88.2)	74 (91.4)	1.0	0.508
	A/A	10 (11.8)	7 (8.6)	0.70 (0.25–2.00)	

Overdominant	G/G‐A/A	45 (59.2)	43 (53.0)	1.0	0.440
	G/A	31 (40.8)	38 (47.0)	1.28 (0.68–2.41)	

Log additive				1.19 (0.71–3.22)	0.602

Control participants vs. DN (Table 11): Similarly, no significant associations were detected for the *TGF-β1* rs1800469 polymorphism when comparing healthy controls (*n* = 76) against patients with T2D and nephropathy (*n* = 47) across any genetic model. For instance, in the codominant model, the G/A genotype had an OR of 1.07 (95% CI: 0.48–2.36, *p* = 0.952), and the A/A genotype had an OR of 0.90 (95% CI: 0.28–2.87, *p* = 0.952). All other models (dominant OR = 1.03, *p* = 0.940; recessive OR = 0.86, *p* = 0.790; overdominant OR = 1.10, *p* = 0.798; log‐additive OR = 1.06, *p* = 0.621) also yielded insignificant results.

**TABLE 11:** Single‐locus analysis to detect the association between *TGF-B1* rs1800469 and Type 2 diabetes with nephropathy in codominant, dominant, recessive, overdominant, and log‐additive modes: crude analysis.

Model	Genotype	Control individuals *N* = 76 *N* (%)	Patients with Type 2 diabetes with nephropathy *N* = 47 *N* (%)	OR (95% CI)	*p* value
Codominant	G/G	36 (47.4)	25 (53.2)	1.0	0.581
	G/A	31 (40.8)	19 (40.4)	0.88 (0.41–1.90)	
	A/A	9 (11.8)	3 (6.4)	0.48 (1.12–1.95)	

Dominant	G/G	36 (47.4)	25 (53.2)	1.0	0.530
	G/A‐A/A	40 (52.6)	22 (46.8)	0.79 (0.38–1.64)	

Recessive	G/G‐G/A	67 (88.2)	44 (93.6)	1.0	0.321
	A/A	9 (11.8)	3 (6.4)	0.51 (0.13–1.98)	

Overdominant	G/G‐A/A	45 (59.2)	28 (59.6)	1.0	0.968
	G/A	31 (40.8)	19 (40.4)	0.99 (0.47–2.07)	

Log additive				0.82 (0.39–2.01)	0.560

#### 3.7.2. TGF‐β1 rs1800470 Analysis

Control participants vs. T2D (Table 12): Analyzing the *TGF-β1* rs1800470 polymorphism revealed no significant associations when comparing healthy controls (*n* = 76) against patients with T2D and without nephropathy (*n* = 81) across any genetic model. In the codominant model, the A/G genotype showed an OR of 1.65 (95% CI: 0.82–3.30, *p* = 0.253), and the G/G genotype showed an OR of 0.89 (95% CI: 0.31–2.57, *p* = 0.253). No significant associations were observed in the dominant (OR = 1.47, *p* = 0.259), recessive (OR = 0.65, *p* = 0.377), overdominant (OR = 1.70, *p* = 0.100), or log‐additive (OR = 1.09, *p* = 0.293) models.

**TABLE 12:** Single‐locus analysis to detect the association between *TGF-B1* rs1800470 and Type 2 diabetes without nephropathy in codominant, dominant, recessive, overdominant, and log‐additive modes: crude analysis.

Model	Genotype	Control individuals *N* = 76 *N* (%)	Patients with Type 2 diabetes without nephropathy *N* = 81 *N* (%)	OR (95% CI)	*p* value
Codominant	G/G	28 (36.8)	23 (28.4)	1.0	0.253
	G/A	37 (48.7)	50 (61.7)	1.65 (0.82–3.30)	
	A/A	11 (14.5)	8 (9.9)	0.89 (0.31–2.57)	

Dominant	G/G	28 (36.8)	23 (28.4)	1.0	0.259
	G/A‐A/A	48 (63.2)	58 (71.6)	1.47 (0.75–2.88)	

Recessive	G/G‐G/A	65 (85.5)	73 (90.1)	1.0	0.377
	A/A	11 (14.5)	8 (9.9)	0.65 (0.25–1.71)	

Overdominant	G/G‐A/A	39 (51.3)	31 (38.3)	1.0	0.100
	G/A	37 (48.7)	50 (61.7)	1.70 (0.90–3.21)	

Log additive				1.09 (0.32–2.95)	0.293

Control participants vs. DN (Table 13): Consistently, no significant associations were observed for the *TGF-β1* rs1800470 polymorphism when comparing healthy controls (*n* = 76) against patients with T2D and nephropathy (*n* = 47) across any of the genetic models. For the codominant model, the A/G genotype showed an OR of 1.07 (95% CI: 0.48–2.36, *p* = 0.952), and the G/G genotype showed an OR of 0.90 (95% CI: 0.28–2.87, *p* = 0.952). The dominant (OR = 1.03, *p* = 0.940), recessive (OR = 0.86, *p* = 0.790), overdominant (OR = 1.10, *p* = 0.798), and log‐additive (OR = 1.06, *p* = 0.621) models also did not yield significant results.

**TABLE 13:** Single‐locus analysis to detect association between *TGF-B1* rs1800470 and Type 2 diabetes with nephropathy in codominant, dominant, recessive, overdominant, and log‐additive modes: crude analysis.

Model	Genotype	Control individuals| *N* = 76 *N* (%)	Patients with Type 2 diabetes with nephropathy *N* = 47 *N* (%)	OR (95% CI)	*p* value
Codominant	G/G	28 (36.8)	17 (36.2)	1.0	0.952
	G/A	37 (48.7)	24 (51.1)	1.07 (0.48–2.36)	
	A/A	11 (14.5)	6 (12.8)	0.90 (0.28–2.87)	

Dominant	G/G	28 (36.8)	17 (36.2)	1.0	0.940
	G/A‐A/A	48 (63.2)	30 (63.8)	1.03 (0.48–2.19)	

Recessive	G/G‐G/A	65 (85.5)	41 (87.2)	1.0	0.790
	A/A	11 (14.5)	6 (12.8)	0.86 (0.30–2.52)	

Overdominant	G/G‐A/A	39 (51.3)	23 (48.9)	1.0	0.798
	G/A	37 (48.7)	24 (51.1)	1.10 (0.53–2.28)	

Log additive				1.06 (0.77–2.33)	0.621

In summary, crude single‐locus analyses demonstrated no significant association between either *TGF-β1* rs1800469 or rs1800470 polymorphisms and the risk of T2D or T2D with nephropathy compared against control, across any of the tested genetic models.

## 4. Discussion

Our comprehensive analysis, including robust statistical adjustments for known confounders, revealed novel insights into the genetic understanding of these complex conditions in this specific ethnic group. TGF‐β1 has been highlighted for its role in fostering renal fibrosis and inflammation in patients with DN. Although this study indicates no significant association between the *TGF-β1* rs1800470 and rs1800469 polymorphisms and T2D, with or without DN, we observed a significant association between the *TGF-β1* rs1800470 polymorphism and albuminuria in DN. Further, we recorded the highest level of albumin in patients with the A/A genotype, while its lowest was recorded in patients with the G/G genotype.

TGF‐β1 is a pleiotropic cytokine widely recognized as a key mediator in DN pathogenesis [21]. It is critical in promoting ECM accumulation, glomerulosclerosis, and tubulointerstitial fibrosis, all of which are pathological processes contributing to renal dysfunction as well as the development and progression of albuminuria [22]. The rs1800470 polymorphism (also known as +869 A > G, or c.29T > C, Leu10Pro) is a coding SNP located in the signal peptide region of the TGF‐β1 protein [23]. Such a change potentially alters the processing, secretion, expression levels, stability, or biological activity of the mature TGF‐β1 protein [24]. The A allele–associated genetic variant may lead to a more profibrotic or proproteinuric form of TGF‐β1, consequently driving increased renal damage and higher albuminuria. Conversely, the G allele–associated variant could be less active, more stable, or lead to altered signaling, thereby mitigating these detrimental effects and contributing to lower albumin excretion. This genotype–phenotype correlation suggests that rs1800470 may not primarily initiate DN but rather act as a crucial genetic modifier that influences the severity or phenotypic presentation of renal damage after DN onset. This concept of genetic modifiers influencing disease severity is increasingly recognized in complex diseases [25].

A primary finding of our study was the lack of a significant association between both *TGF-β1* rs1800469 and rs1800470 genotypes and the presence of T2D (either with or without nephropathy) compared against that of the control. Furthermore, these SNPs did not differentiate between patients with diabetes and with and without nephropathy. These findings remained consistent even after adjusting for critical confounding factors such as age, BMI, dyslipidemia, and systolic blood pressure (Tables 3, 5, and 7). This absence of association contrasts with some previous studies that implicated *TGF-β1* polymorphisms in T2D susceptibility or progression to DN in other populations [9, 16, 19, 26]. Certain variants of TGF‐β1 have been linked to an increased risk of T2D or a faster decline in renal function in Caucasian and Asian cohorts [27, 28]. However, our results align with other studies that have reported no significant associations in different ethnic groups, suggesting that the genetic impact of these particular SNPs on T2D and DN may be population‐specific due to varying genetic backgrounds and environmental interactions [16, 29–32]. The specific allele frequencies and linkage disequilibrium patterns within the Saudi population might differ from those observed in other ancestries, potentially modifying the phenotypic effects of these variants. Population differences in DN and its risk factors require investigation through ethnicity‐specific association studies.

Despite the general lack of association with disease status, our research revealed a notable link between the *TGF-β1* rs1800470 genotype and albumin levels in patients with diabetes suffering from nephropathy (*p* = 0.002, Table 9). Specifically, individuals carrying the A/A genotype for rs1800470 exhibited the highest albumin levels, whereas those with the G/G genotype had the lowest. This finding is particularly intriguing considering TGF‐β1’s well‐established role as a key profibrotic cytokine involved in the pathogenesis of kidney diseases, including DN [33]. TGF‐β1 signaling dysregulation can lead to ECM accumulation, glomerular sclerosis, and tubular atrophy—processes that are central to albuminuria development, a hallmark of DN [34]. Lower albumin levels in patients with nephropathy often indicate more severe renal damage and compromised glomerular filtration barrier integrity. Therefore, our observation suggests that the G/G genotype of rs1800470 might be associated with a more pronounced decline in renal function, as evidenced by lower albumin levels, potentially reflecting a more detrimental effect on kidney health in this context. Conversely, the A/A genotype is associated with better‐preserved albumin levels, hinting at a protective or less damaging influence. This finding warrants further investigation into the functional implications of the rs1800470 polymorphism on *TGF-β1* expression, protein activity, or downstream signaling pathways that affect albumin excretion.

Our study found no significant association between the *TGF-β1* rs1800469 polymorphism and T2D with or without DN in Saudi patients. This result disagrees with a previous study conducted on 172 Iraqi patients with DM, where *TGF-β1* rs1800469 polymorphisms were significantly different in genotype distribution and allelic frequencies between patients with T2D and control participants and were associated with clinical characteristics. Thus, this SNP was related to T2D susceptibility [35]. Our results agree with those of Raina et al., a study conducted on the Indian population found that the TT (AA) genotype provided a 5.5‐fold risk toward ESRD cases from Jammu and Kashmir and no risk for cases from Punjab [19].

A significant strength of our study lies in its rigorous methodology, including the careful stratification of participant groups, allowing for precise comparisons among the control participants and patients with diabetes and with and without nephropathy. The application of multivariate logistic regression to adjust for key confounding factors, such as age, BMI, dyslipidemia, and systolic blood pressure, enhances data reliability. Furthermore, the power analysis confirmed that our sample size was adequate to detect small‐to‐medium effect sizes, providing confidence in both the observed associations and nonassociations. Utilizing Sanger sequencing validated our genotyping results, ensuring the accuracy and integrity of our genetic data.

However, this study has certain limitations. The case–control design inherently limits our ability to establish causality or track the longitudinal progression of albuminuria in direct relation to genotypes. A relatively modest sample size for the DN group (*n* = 47), while sufficient to detect the observed association, suggests that the findings would benefit from replication in larger, independent cohorts. Additionally, while we controlled for several important clinical and demographic factors, other unmeasured genetic or environmental factors could also influence albumin levels and potentially interact with *TGF-β1* genotypes.

Future research should prioritize functional studies to thoroughly elucidate the biological mechanisms whereby the rs1800470 polymorphism—specifically, the Leu10Pro substitution—affects *TGF-β1* expression, secretion, activity, and downstream signaling pathways related to renal injury and albuminuria. Large‐scale, prospective longitudinal studies involving diverse diabetic populations are essential to validate our findings, evaluate the rs1800470 genotype as a potential prognostic marker for DN progression, and investigate its role in personalized therapeutic strategies for managing albuminuria. Additionally, exploring other SNPs within the TGF‐β1 pathway or interacting genes could yield a more comprehensive understanding of the complex genetic architecture of DN pathogenesis and its associated phenotypic variability. Future studies may also consider comparing our allele frequencies with existing regional genomic databases to provide broader context for our findings.

## 5. Conclusions

Our data suggest that *TGF-β1* rs1800470 acts as a phenotypic modifier of albuminuria severity in patients with established DN, rather than indicating a causal role in disease onset or serving as a standalone predictive biomarker. Functional studies (expression and protein assays) and longitudinal clinical validation are required to clarify its mechanistic impact and to evaluate any prognostic or therapeutic utility.

## Funding

Open‐access publishing was facilitated by the Deanship of Scientific Research (DSR) at King Abdulaziz University, as part of the Wiley–King Abdulaziz University agreement.

No funding was received for this manuscript.

## Conflicts of Interest

The authors declare no conflicts of interest,

## Data Availability

All data generated or analyzed during this study are included in this published article.
